# Atrioventricular Dissociation following Blunt Chest Trauma

**DOI:** 10.1155/2014/349652

**Published:** 2014-03-27

**Authors:** Salim Surani, Karen Allen, Cynthia Ocegueda-Pacheco, Joseph Varon

**Affiliations:** ^1^Texas A&M University, Texas Corpus Christi, 1177 West Wheeler Avenue, Suite 1, Aransas Pass, TX 78336, USA; ^2^Bay Area Medical Center, University of North Texas, 7101 S Padre Island Dr, Corpus Christi, TX 78412, USA; ^3^Hospital Zambrano Monterrey, San Patricio 112, Real San Agustin, 66278 San Pedro Garza Garcia, NL, Mexico; ^4^The University of Texas Health Science Center, University General Hospital, 7501 Fannin Street, Houston, TX 77054, USA

## Abstract

Blunt chest trauma (BCT) is a common clinical presentation seen in emergency departments. Few cases of cardiac conduction abnormalities due to BCT have been reported in the medical literature. This dysrhythmias may present as permanent conduction defects requiring permanent pacemaker or may have temporary conduction abnormalities requiring temporary pacemaker or supportive care. We present the case of a young woman who suffered from BCT after being kicked by a horse with the development of a significant substernal hematoma. She developed temporary atrioventricular block, which was completely resolved with the decrease in the size of the substernal hematoma suffered.

## 1. Introduction

Blunt cardiac injury (BCI) inducing cardiac conduction abnormalities is not a common medical occurrence and probably underreported in the medical literature. An initial review by Dolara and Pozzi in 1966 presented 23 cases of atrioventricular block (AVB) from BCI [1]. Two decades later, Carr and coworkers reported a transient bifascicular block after blunt injury [2]. We present the case of a woman with no previously known coronary artery disease or dysrhythmia, who developed a self-limited complete atrioventricular nodal block after sustaining blunt chest trauma (BCT) due to a horse kick injury to the chest.

## 2. Case Presentation

**Figure 1:**
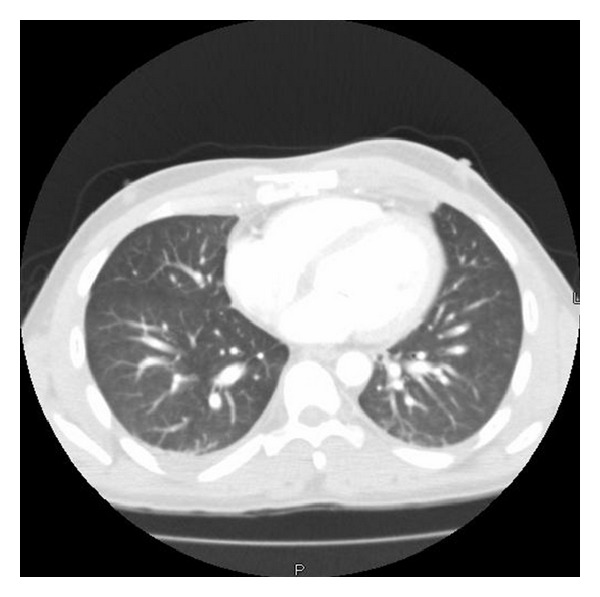
Chest CT revealing a sternal fracture and a small substernal hematoma.

A 25-year-old woman with no past medical or surgical history was brought to the Emergency Department (ED) after being kicked by a horse in her chest. The patient was found to have a midsternal fracture with underlying hematoma on computed tomography (CT) chest (see Figure 1). Her blood pressure was 102/67 mm hg, her heart rate was 58/min, and her respiratory rate was 18/min, and oxygen saturation was 99% while breathing room air. Her white blood cell count was normal and her hemoglobin was 14.1 gm/L. The creatinine phosphokinase 494/L (normal 38/L–174/L) and troponin level was 3.74 *μ*g/L (0.00–0.08 *μ*g/L). These were felt to be secondary to a blunt cardiac contusion. Her elevated cardiac enzymes reverted back to normal within 48 hours.

**Figure 2:**
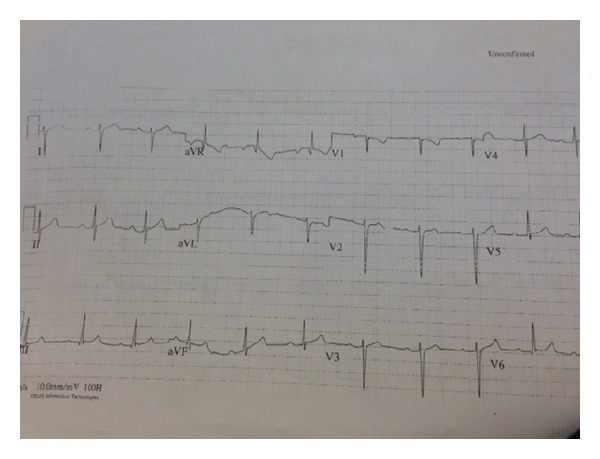
EKG revealing sinus rhythm at a rate of 65 bpm with first-degree AV block and left anterior hemiblock.

**Figure 3:**
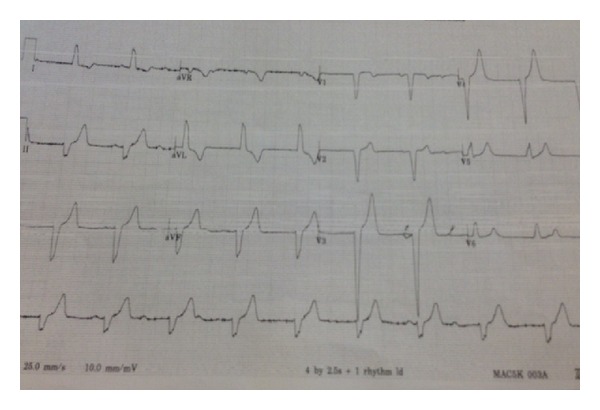
Followup EKG revealing complete AV block along with left bundle branch block and a ventricular rate of ~55 bpm.

**Figure 4:**
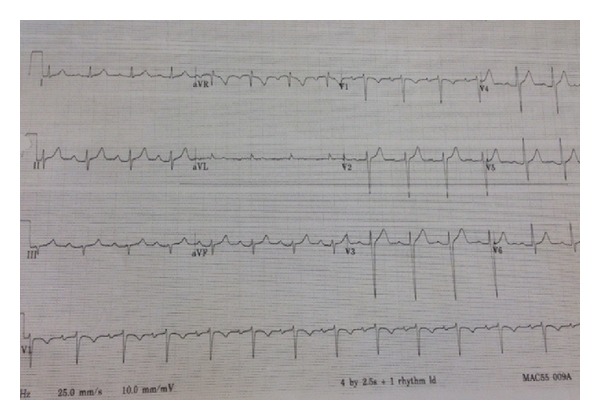
EKG showing sinus rhythm with resolution of the complete AV block and depicting sinus rhythm at a rate of 85 bpm with first-degree AV block and left anterior hemiblock.

**Figure 5:**
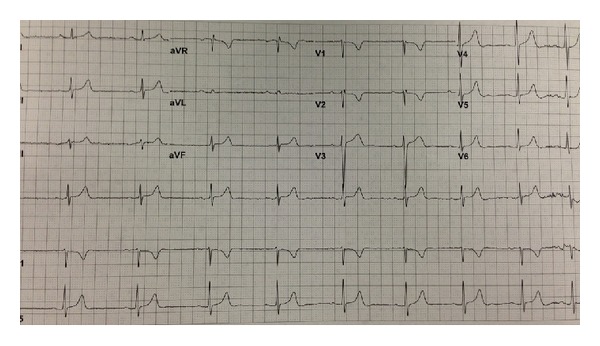
EKG showing sinus rhythm with rate of 56 bpm and PR interval of 180 ms.

The patient's electrocardiogram (EKG) on admission revealed normal sinus rhythm with rate of 65 bpm (beats per minute) with first-degree atrioventricular block (AV Block) and left anterior hemiblock as depicted in Figure 2. The patient was admitted to the coronary care unit (CCU) for observation. 13 hours after her injury, she developed a third-degree AV block along with left bundle branch block with the rate of 55 bpm (see Figure 3). At that point, she was totally asymptomatic with normal hemodynamic parameters. A cardiology consultant was obtained and felt that the patient's AVB was likely secondary to the substernal hematoma causing mechanical compression of the AV bundle. A 2D echocardiogram was normal with no evidence of cardiac injury with a normal with an ejection fraction of 70%. She was kept in CCU and within seventy-two hours, her rhythm converted to normal sinus rhythm with rate of 85 bpm and first-degree AV block and left anterior hemiblock as depicted in Figure 4. She was discharged home in a stable condition with an outpatient followup with her primary care physician. A four-week followup revealed that the EKG is in normal sinus rhythm with the rate of 56 bpm (see Figure 5).

## 3. Discussion

According to the American Association for the Surgery of Trauma, the effects of BCI can range from being asymptomatic with minor EKG abnormalities to rupture of the cardiac anatomy, heart failure, and even traumatic avulsion of the heart [3]. Conduction abnormalities, both transient and permanent, are documented throughout the literature. Most recent reports of complete AVB in this setting indicate a predominance of permanent AV block, requiring pacemaker placement [4]. Our patient was fortunate that while her AV block developed quickly after trauma, her conduction deficit was temporary and self-limited, requiring only supportive measures. There have been a series of reports that have documented a late occurrence of these abnormalities. For example, Lazaros and coworkers reported a delayed development of a complete heart block [5]. This “delayed” development of complications in their case was theorized to originate from fibrosis or scarring around the AV node and the conduction system.

Most conduction abnormalities are postulated to arise from myocardial infarction or ischemia, stunning of the conduction system, or excessive cholinergic activation [6]. Basic advance life support protocols should be followed when managing patients with BCI, including the concept that any tachycardia should be presumed secondary to hemorrhage or volume loss until proven otherwise [5, 7]. Patients with BCI should be considered as high priority for the transthoracic echocardiogram in the ED to evaluate for potential pericardial hemorrhage and valvular dysfunction [5, 7]. Cardiac enzymes should be monitored at regular intervals to rule out traumatic myocardial infarction.

## 4. Conclusions

Dysrhythmias (including AVB) following blunt chest wall injury can be self-limited or may require permanent pacemaker. Despite the resolution of our patient's AVB, most sources suggest close observation and followup in case delayed complications arise.
